# Updates and Comparative Analysis of the Mitochondrial Genomes of *Paracoccidioides* spp. Using Oxford Nanopore MinION Sequencing

**DOI:** 10.3389/fmicb.2020.01751

**Published:** 2020-08-04

**Authors:** Elizabeth Misas, Oscar M. Gómez, Vanessa Botero, José F. Muñoz, Marcus M. Teixeira, Juan E. Gallo, Oliver K. Clay, Juan G. McEwen

**Affiliations:** ^1^Cellular and Molecular Biology Unit, Corporación para Investigaciones Biológicas, Medellín, Colombia; ^2^Colombia Wisconsin One Health Consortium, Universidad Nacional de Colombia, Medellín, Colombia; ^3^Genoma CES, Universidad CES, Medellín, Colombia; ^4^Broad Institute of MIT and Harvard, Cambridge, MA, United States; ^5^Faculty of Medicine, University of Brasília, Brasília, Brazil; ^6^Translational Microbiology and Emerging Diseases (MICROS), School of Medicine and Health Sciences, Universidad del Rosario, Bogotá, Colombia; ^7^School of Medicine, Universidad de Antioquia, Medellín, Colombia

**Keywords:** mitochondria, genome, *Paracoccidioides*, Oxford Nanopore, NGS

## Abstract

The mitochondrial genome of the *Paracoccidioides brasiliensis* reference isolate Pb18 was first sequenced and described by Cardoso et al. (2007), as a circular genome with a size of 71.3 kb and containing 14 protein coding genes, 25 tRNAs, and the large and small subunits of ribosomal RNA. Later in 2011, Desjardins et al. (2011) obtained partial assemblies of mitochondrial genomes of *P. lutzii* (Pb01), *P. americana* (Pb03), and *P*. *brasiliensis sensu stricto* (Pb18), although with a size of only 43.1 kb for Pb18. Sequencing errors or other limitations resulting from earlier technologies, and the advantages of NGS (short and long reads), prompted us to improve and update the mtDNA sequences and annotations of two *Paracoccidioides* species. Using Oxford Nanopore and Illumina read sequencing, we generated high-quality complete *de novo* mitochondrial genome assemblies and annotations for *P. brasiliensis* (Pb18) and *P. americana* (Pb03). Both assemblies were characterized by an unusually long spacer or intron region (>50 kb) between exons 2 and 3 of the nad5 gene, which was moderately conserved between Pb03 and Pb18 but not similar to other reported sequences, except for an unassigned contig in the 2011 assembly of Pb03. The reliability of the insert missing from previous mtDNA genome assemblies was confirmed by inspection of the individual Nanopore read sequences containing nad5 coding DNA, and experimentally by PCR for Pb18. We propose that the insert may aid replication initiation and may be excised to produce a smaller structural variant. The updated mtDNA genomes should enable more accurate SNP and other comparative or evolutionary analyses and primer/probe designs. A comparative analysis of the mtDNA from 32 isolates of *Paracoccidioides* spp., using the SNPs of the aligned mitochondrial genomes, showed groupings within the *brasiliensis* species complex that were largely consistent with previous findings from only five mitochondrial loci.

## Introduction

*Paracoccidioides* spp. is a thermal dimorphic fungus, pathogenic for humans, which at temperatures below 24°C grows as mycelium in the environment and at a temperature of 37°C grows as yeast in a mammalian host or *in vitro*. *Paracoccidioides* spp. is the causative agent of paracoccidioidomycosis (PCM), a systemic mycosis that affects the population of Latin America (Brummer et al., 1993).

In the genus *Paracoccidioides*, five species have been described, *P. lutzii, P. brasiliensis* (S1), *P. americana* (PS2), *P. restrepiensis* (PS3), and *P. venezuelensis* (PS4). The last four constitute the *brasiliensis* species complex. Each of the *Paracoccidioides* species can be identified using molecular and morphological criteria (Turissini et al., 2017), but the grouping of the species within the *brasiliensis* complex has not been entirely consistent across nuclear and mitochondrial studies.

Mitochondrial genome assemblies from whole genome sequencing projects are currently available as draft sequences for three strains of the genus *Paracoccidioides*, two isolates of the *brasiliensis* species complex, *P. brasiliensis* (Pb18) and *P. americana* (Pb03), and one representative isolate of the species *P. lutzii* (Pb01) (Desjardins et al., 2011). For isolate Pb18, there is also available an independent, earlier assembly and annotation by Cardoso et al. (2007) (GenBank: AY955840). Unlike the draft sequences mentioned previously, which are partial, the annotated assembly Pb18 AY955840 covers almost all of the mitochondrial gene content and was used in this work as a reference.

In the work of Cardoso et al. (2007) the mitochondrial genome was obtained by physical separation of the mitochondria followed by Sanger sequencing; said genome was described as a circular molecule of 71.3 kb with 14 sequences of protein coding genes. By contrast, in the work of Desjardins et al. (2011) the nuclear and partial mitochondrial genomes of three strains of *Paracoccidioides* spp. (Pb18, Pb03, and Pb01) were sequenced together using Sanger technology. These genomes were assembled with Arachne and the mitochondrial scaffolds were identified and separated *in silico*. For the Pb18 isolate, four supercontigs with a total size of only 43.12 kb were reported, that is, covering only 60.4% of the mitochondrial genome reported by Cardoso et al. (2007).

In the present work, we re-sequenced the DNA with Oxford Nanopore and Illumina to obtain improved whole mitochondrial genome sequences and annotations for strains Pb18 and Pb03. We used the SPAdes program, which implements a *de novo* hybrid assembly strategy that combines both types of sequencing.

In addition to long and short read sequencing of the two reference isolates Pb18 and Pb03, we also used Illumina technology to sequence four isolates of *P. lutzii* and 28 isolates of the *brasiliensis* species complex from four countries, Brazil, Colombia, Argentina, and Venezuela, thus covering a large part of the geographic distribution of *Paracoccidioides* spp. (Muñoz et al., 2016).

The reads of all of these isolates were included in the present work to identify the polymorphisms present in the mitochondrial genome of *Paracoccidioides* and to determine, using the new data, the grouping of the isolates from the *brasiliensis* species complex that are indicated by the mitochondrial sequences.

## Materials and Methods

### Sampling, DNA Extraction, and WGS

Thirty-four isolates of the *Paracoccidioides* genus of different geographic origins were included in this study, namely, 20 isolates from Brazil, 6 isolates from Colombia, 3 isolates from Argentina, and 5 isolates from Venezuela (Muñoz et al., 2016).

The selected isolates were distributed in the phylogenetic species as follows: 15 from *P. brasiliensis* (11 from S1a and 4 from S1b), 7 isolates from *P. restrepiensis* (PS3), 4 from *P. americana* (PS2), 4 from *P. venezuelensis* (PS4), and 4 from *P. lutzii* (Supplementary Table S1). PbT1F1, T15N1, PbCAB, Pb337, and PbT10B1 were isolated from armadillo, Pb300 was isolated from the soil, Pb262 was isolated from dog food, and the others were clinical isolates from patients with PCM (Supplementary Table S1).

The DNA was extracted using the phenol-chloroform method (Diez et al., 1999) with some modifications. Before the treatment with organic solvents, the biomass was macerated with liquid nitrogen. Library preparation and sequencing was carried out in three sequencing centers (University of California at Berkeley, University of Illinois Urbana–Champaign, Broad Institute) following the protocols proposed by Illumina in the guide “Preparing Samples for Sequencing Genomic DNA^1^.”

All isolates were sequenced using Illumina HiSeq2500 platforms. In addition, the Pb18 and Pb03 isolates, representing the reference strains for *P. brasiliensis* and *P. americana*, respectively, were sequenced using Oxford Nanopore technology R9.5 flowcells at the University of Illinois Urbana–Champaign.

### Assembly and Annotation of the Mitochondrial Genomes of *P. brasiliensis* (Pb18) and *P*. *americana* (Pb03) Using Oxford Nanopore Reads

Two *de novo* assembly programs, SPAdes v-3.10 (Bankevich et al., 2012) and Canu v-1.5 (Koren et al., 2017), were used to obtain complete genome assemblies (nuclear and mitochondrial scaffolds) for the isolates Pb18 and Pb03. The Oxford Nanopore reads were assembled using Canu v-1.5, whereas SPAdes v-3.10 allowed the construction of a hybrid assembly in a single run including both the Illumina and Oxford Nanopore reads (Supplementary Table S2).

Subsequently, the contigs corresponding to the mitochondrial sequences were identified and extracted from each assembly. After comparing them with the available draft genome sequences, the mitochondrial contig assembled by SPAdes v-3.10, i.e., incorporating the contiguity information from the Oxford Nanopore long reads, was selected for each of the two isolates. The Canu v-1.5 contigs served to confirm that each of the two assemblies obtained via SPAdes v-3.10 represented a complete mtDNA genome because the repeated sequences at the ends of the selected mitochondrial contig, identified using MUMmer v-3.23, could be joined to circularize the sequence, which then had the same extent as the corresponding SPAdes assembly. Finally, the sequence was used as a reference to map the Illumina reads with BWA v-0.6.1 (Li and Durbin, 2009).

In the mitochondrial contigs of both Pb03 and Pb18, a region was observed that had been absent in the previously available assemblies. This “insert” was consistently found both in assemblies obtained by SPAdes v-3.10 and in those obtained by Canu v-1.5. We used BLAST-ncbi, EMBOSS isochore for GC plots, and remapping of the reads to characterize this insert.

The annotation of the mitochondrial assemblies for Pb03 and Pb18 was performed via homology based on tBLASTn (Boratyn et al., 2013), using as a basis the previously curated annotation of Cardoso et al. (2007) for the mitochondrial genome of Pb18 (E. Misas, M.Sc. thesis) except in the case of its apparently incomplete gene *nad5*, where we used an annotation of *Neurospora crassa* (KC683708.1) as reference. tRNA coordinates were predicted using tRNAScan-SE^2^ (Chan and Lowe, 2019). As a check, we then used MFannot^3^ (Lang et al., 2007) to automatically draft-annotate all genes except *nad5* (which was split by the boundaries of the linear sequence), and manually compared and clarified any inconsistencies.

### Analyses of SNPs Identified in the Whole Mitochondrial Genomes of the *Paracoccidioides* Genus

For the 33 isolates of the genus *Paracoccidioides*, the reference assembly program BWA v-0.5.9 was run with default parameters, using as reference the obtained mitochondrial genome sequence of Pb18 assembled by SPAdes v-3.10.

From the resulting BWA alignments, the Pilon v-1.6 program was run with the “-variant” parameter, which generated a report of SNPs for each of the 33 isolates in the ^*^.vcf (Variant Call Format) format.

To build a phylogenetic tree with the information of the mitochondrial SNPs, the variable positions in Fasta format were recovered from the.vcf files generated by Pilon v-1.6 using a Perl script. To consider a position as a variable, we required that it should have a minimum coverage of 4 reads and be variable in at least one of the 32 isolates. The resulting sequences were aligned with the ClustalW v-2.1 program, and maximum-likelihood phylogenies were constructed using IQ-Tree v-1.4.4 program (Nguyen et al., 2015) using the TVM nucleotide substitution model and bootstrap analysis based on 1000 replicates.

## Results

### WGS and Assembly of the Mitochondrial Genomes of *P*. *brasiliensis* (Pb18) and *P*. *americana* (Pb03)

For Pb18, we obtained 36,126 long reads with an average size of 2.6 kb, and for Pb03 we obtained 71,528 long reads with an average size of 3.1 kb, using MinION. The Illumina HiSeq2500 paired-end short read sequencing produced 46.8 million pairs of reads with a length of 101 bp for the Pb18 strain, and 62 million 101 bp read pairs for the Pb03 strain. The quality of the short reads was evaluated using FastQC v0.11.2 (Brown et al., 2017) and for the Oxford Nanopore long reads of the Pb18 strain, length histograms of the total reads and of the reads mapped to the mitochondrial genome were constructed.

Although generally large differences were observed in the *de novo* assemblies obtained with Canu v-1.5 and SPAdes v-3.10, the recovered mitochondrial contig sequences obtained using the two programs were very similar.

**FIGURE 1:**
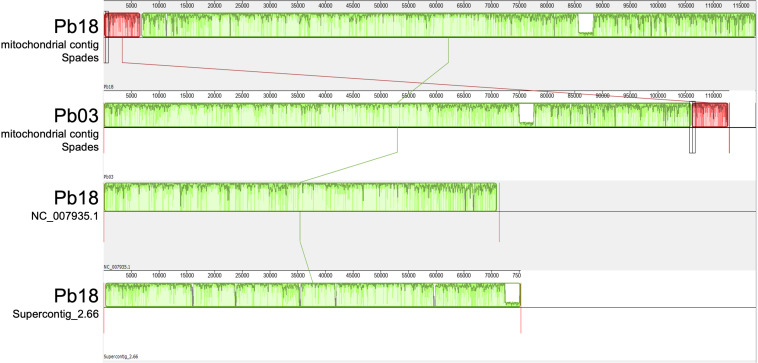
Mauve alignment of present and previous Pb18 and Pb03 assemblies. The top two tracks present the mitochondrial contigs assembled by the SPAdes v-3.10 program in this study, for the Pb18 and Pb03 strains. The bottom two tracks represent the previously available mitochondrial genome draft assemblies for Pb18 (NC_007935.1) reported by Cardoso et al. (2007) and for Pb03 (Supercontig_2.66) reported by Desjardins et al. (2011). Green and red boxes represent Locally Collinear Blocks (LCBs), i.e., conserved segments that appear to be internally free from genome rearrangements. The order of the LCBs is given by the original coordinates of the sequences specified by the assembly program and the coordinates in the previously reported sequences when they are presented as a linear sequence. The plot inside the boxes represents the local quality of the alignment.

**FIGURE 2:**
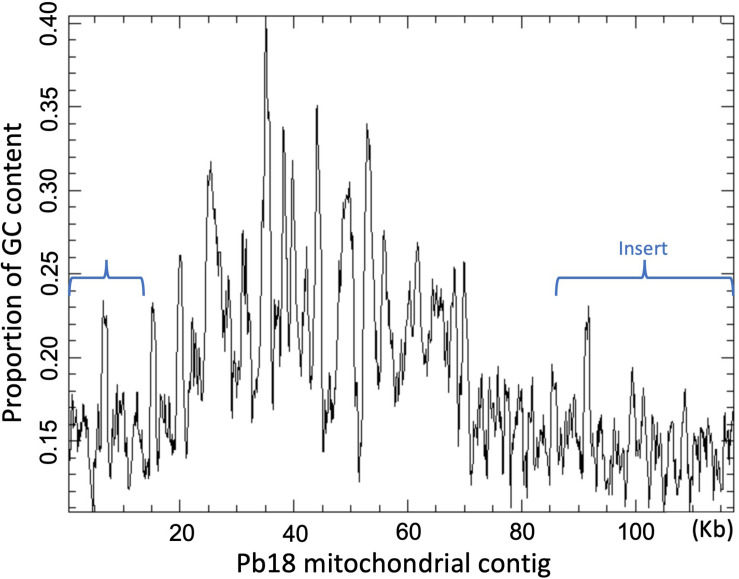
GC plot of the mitochondrial contig of Pb18 assembled by SPAdes v-3.10. The brackets mark the region that is absent in the sequence previously reported by Cardoso et al. (2007).

The SPAdes v-3.10 assembler allowed the construction of a single contig corresponding to the whole mitochondrial genome of the strains Pb18 and Pb03, although in both cases the contig was longer than expected on the basis of previous assemblies. In both mitochondrial sequences, a region, or “insert,” was identified that had been absent in all previously reported mitochondrial genome sequences. This “insert” region, defined as the segment that could not be matched to the previous reference genome of Pb18 or Pb03, respectively, had a length of 47 kb in Pb18 and 38 kb in Pb03 (Figure 1), and a lower percentage of GC than the rest of the contig (Figure 2). The insert sequence of Pb03 is highly similar (> 80% identity) to that of Pb18 throughout its length, and these inserts appear to contain no genes, except for a part of *nad5* exon 2 that had been truncated in the Pb18 reference assembly of Cardoso et al. (2007).

The presence of the insert region was then experimentally confirmed by PCR amplification of two regions near the boundaries of the insert in the Pb18 species of *Paracoccidioides* (Supplementary Material). The two amplicon sequences, both of which amplified successfully in Pb18, were then found by BLASTn to be present also in the new assembly of Pb03, but the Pb18 primers for one of the amplicons did not match precisely in Pb03, and indeed only one of the two amplicons was experimentally observed in that species (Supplementary Material).

A second check that the insert was not just an assembly artifact was done by searching the Oxford Nanopore long reads of Pb03 for individual long reads that contained coding DNA from exons 2 or 3 of nad5, and then inspecting the sequence-level alignment of the six longest reads with high-quality tBLASTn matches to *nad5* obtained on the exon 2 end (total alignment length 5423 nt). The contiguous long sequence reads all corresponded to the full genome assembly for Pb03 we report here. Furthermore, this observation suggests that in the sample we sequenced, no appreciable structural heteroplasmy (e.g., a structural variant corresponding to the assembly, plus another more compact alternative variant) was present; this observation does not, however, exclude that such heteroplasmy may be present in other conditions *in vivo*.

### Annotation

**FIGURE 3:**
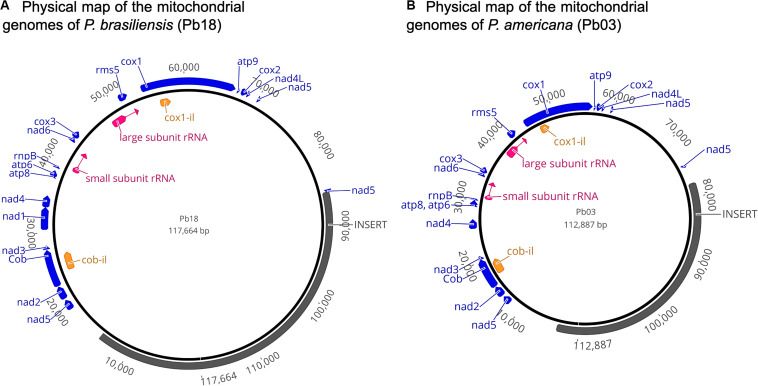
**(A,B)** Physical map of the mitochondrial genomes of *P. brasiliensis* (Pb18 and Pb03). Protein coding genes are shown in blue, rRNAs are shown in pink, and ORFs in introns are shown in orange. The regions that were absent in the sequences previously reported are annotated as “insert” and allow comparison with previous studies but do not represent a biological feature of the sequence.

In the mitochondrial genome assembly of the Pb18 strain, the 14 protein coding genes were identified, in the following order: NAD dehydrogenase subunit 2 (*nad2*), apocytochrome b (*cob*), NAD dehydrogenase subunits 3 (*nad3*), 1 (*nad1*), and 4 (*nad4*), ATP synthase subunits 8 (*atp8*) and 6 (*atp6*), NAD dehydrogenase subunit 6 (*nad6*), cytochromoxidase subunit 3 (*cox3*), ribosomal protein rms5 (*rms5*), cytochromoxidase subunit 1 (*cox1*), ATP synthase subunit 9 (*atp9*), cytochromoxidase subunit 2 (*cox2*), and NAD dehydrogenase subunits 4l (*nad4l*) and 5 (*nad5*) (Figure 3).

Some of these genes contain introns, e.g., in the sequence of the cob gene, two introns were identified, and one of them was recognized as containing a part of *cob-il*, which spans the sequence of *cob* exon 2 and intron 2. The *nad1* gene has one intron. In the *nad5* gene, three exons were identified. Finally, in the sequence of the *cox1* gene, eight exons were identified, and the intron located between exons 1 and 2 was annotated as *cox1-il*. The two subunits of ribosomal RNA were identified by BLASTn and 25 tRNAs.

In the annotation of the assembly of the mitochondrial genome of Pb03, the genes conserve the same order. Also, an additional protein with function laglidadg endonuclease was identified between exons 2 and 3 of the *cox1* gene.

### Analyses of SNPs Identified in the Whole Mitochondrial Genome of the *Paracoccidioides* Genus

Read data were aligned by BWA v-0.5.9 using as reference the region 14517-85557 of the mitochondrial contig of the isolate Pb18 from Brazil we had obtained via Oxford Nanopore sequencing after excluding the insert region. Because the sequence of the insert is highly AT rich, we decided to exclude it to avoid bias in the process of calling SNPs coming from low-quality mapping of short reads. Single-nucleotide polymorphism (SNP) variants were identified using Pilon v-1.6. The mean coverage of the mapped reads for the 32 isolates was 4165×. The isolates PbED01 and Pb1578 were excluded from the phylogenetic analysis because they presented very low coverage, 12× and 19×, respectively (Supplementary Table S1).

**FIGURE 4:**
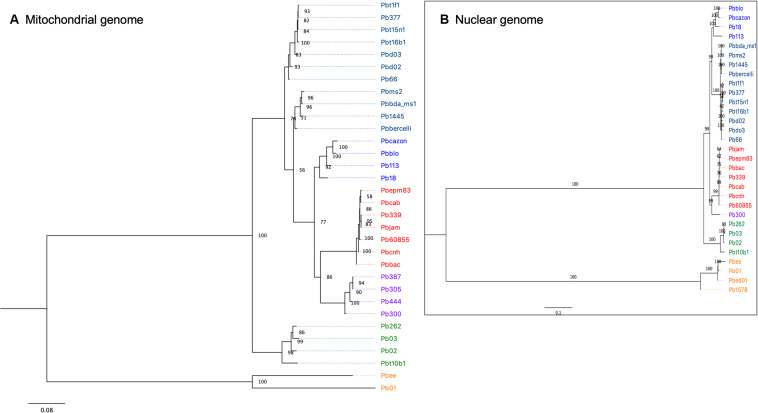
**(A)** Maximum-likelihood phylogeny (with model TMV) obtained using IQ-tree v.1.4.4. The final data set included a total of 1294 nucleotide sites for 32 isolates of the *Paracoccidioides* genus. Bootstrap values calculated using 1000 reiterations are shown. **(B)** Maximum-likelihood phylogeny constructed with RAxML, using 614,570 nuclear SNPs.

For the phylogenetic reconstruction, 1294 variable positions were used. At least one strain in each of these positions had a variant and the minimum coverage was four reads. In the phylogenetic tree, seven clusters are observed, of which four correspond to the described phylogenetic species. The cluster that corresponds to the species *P. lutzii* is the one that presents the greatest number of changes with respect to the rest, and in particular the isolates of Pb01 and PbEE have accumulated many differences in their mitochondrial genomes (Figure 4).

The phylogenetic tree for the *brasiliensis* species complex succeeds in grouping the seven isolates of the species *P. restrepiensis* (PS3), which all grouped in the same cluster. Another cluster corresponds to *P. americana*, where the four isolates of this species were grouped together. The *P. americana* species shows a greater number of changes than other species in the *brasiliensis* species complex.

The isolates corresponding to *P. brasiliensis* (S1a and S1b) are separated into three distinct groups (Figure 4). The isolates PbCazon, PbBLO, Pb113, and Pb18 form a single clade suggesting monophyly. These four isolates also clustered in a previous analysis of whole nuclear genome SNPs by Muñoz et al. (2016), where the cluster was named S1b. Indeed, that study of nuclear genomes showed two internal groups of S1, i.e., S1b and a clade formed by other S1 isolates, called S1a.

## Discussion

### Updated and Annotated Mitochondrial Genome Sequences for Pb18 and Pb03

The mitochondrial genomes of some fungi can be surprisingly difficult to assemble *de novo*, despite their small size, which is generally less than 100 kb (Muñoz et al., 2014). The fungal mitochondrial genome may contain a high number of repeated regions, which may hinder the process of *de novo* assembly, as we have discussed previously (Muñoz et al., 2014; Misas et al., 2016). At the (≤17 bp) oligonucleotide level, *Paracoccidioides* has one of the most pronounced repeat abundances of the fungal genera we compared in Misas et al. (2016), where the abundant repeats are furthermore dominated by those consisting only of A and T. The difficulty of assembling fungal mitochondrial genomes is not limited to the use of NGS sequencing. With previous sequencing technologies, problems were reported in earlier attempts to obtain mitochondrial assemblies of high quality for the model yeast *S. cerevisiae* (Foury et al., 1998). The difficulty for *Paracoccidioides* is apparently reflected in the large gaps present in the partial mitochondrial assemblies that were available for all three strains, Pb18, Pb03, and Pb01, and that were obtained as part of a whole genome project using Sanger sequencing (Desjardins et al., 2011), where for example a region containing a low-complexity, pyrimidine-rich segment (between *nad3* and *nad1*) that is present in the previous sequence of Pb18 (Cardoso et al., 2007) and in our new assemblies seems to have been particularly hard to bridge.

We achieved the assembly of the complete mitochondrial genome in a single contig, using a strategy that involves long and short read sequencing. Recently, the assembly of the mtDNA genome of *S. cerevisiae* S288C using Oxford Nanopore sequencing for *de novo* assembly and Illumina/Pilon for fine tuning was reported (Istace et al., 2017), and we also followed a methodology that integrates information from both long and short reads.

### The Long Second Intron or Spacer of the *nad5* Gene

An unexpected finding in both of our *de novo* mitochondrial genome assemblies, those of *Paracoccidioides* strains Pb18 (*brasiliensis* complex) and Pb03 (*P. americana*), was a large insertion of largely repetitive sequences without any encoded gene product, located between the second and third exon of the gene for nad5. So far, the longest sequenced fungal intron located at this position in the coding sequence of *nad5*, corresponding to position 217 in the nad5 protein sequence of *Neurospora crassa* (Zubaer et al., 2019), appears to have been 5513 bp, in the mitochondrial genome of *Endoconidiophora resinifera* [Helotiales; (Zubaer et al., 2018)]. By contrast, the introns or spacers at the corresponding position in both Pb18 and Pb03 are an order of magnitude longer, namely 51,431 bp for Pb18 and 49,922 bp for Pb03.

The intron or intragenic, inter-exon spacer of about 50 kb we observe, exhibiting moderate sequence similarity along the entire spacer between Pb03 and Pb18, appears at first sight to be unlike other *nad5* introns (Zubaer et al., 2019), and indeed only its first few kilobases are recognized as (group IB) intronic DNA by RNAweasel (Lang et al., 2007). Despite various searches of the long intron, we could not unambiguously identify any known elements such as ORFs (using NCBI ORFfinder) with similarity to known proteins, repetitive elements (e.g., Censor reported matches of very AT-rich segments to elements such as Gypsy-1B_CaPs-I and Gypsy-2_PaPs-I but with mismatches at almost all of the few G’s and C’s), or G-quadruplexes as suggested by the visible clusters of G’s (regions reported by QGRS Mapper or imgqfinder appeared to be chance matches to criteria in some of the many purine-only tracts of the intron). However, already *nad5* can present surprises (in addition to its absence together with other complex I genes in some species, including but not limited to the Saccharomycetaceae; Freel et al., 2015), such as an intron of length 4411 bp at the same position, that harbors a classic protein-coding gene, *cox3*, in the fungus *Flammulina velutipes* (Basidiomycota/Agaricales; Zubaer et al., 2019). More generally, other surprises or spontaneous genome transconformations are not uncommon in intergenic DNA of fungal mitochondrial genomes and in some intronic DNA (Bernardi, 2005, p. 21–48).

In this wider context of large expanses of intergenic or non-coding fungal mtDNA, the region between exons 2 and 3 of our Pb18 and Pb03 assemblies appears to follow a classic pattern of long, extremely GC-poor segments (where the complexity reduces almost to that of a two-letter alphabet, A and T) alternating with much shorter, GC-richer clusters. Such a scenario leads, in some species including *Saccharomyces cerevisiae*, to a landscape of secondary structures (in some cases temperature dependent; Bernardi, 2005, p. 42), where copies (repeats) of certain oligonucleotides can occur at high frequencies and also open the possibility of out-of-register recombination between similar sequence motifs, leading to occasional excision of circularizable subsequences or subgenomes. In the case of *S. cerevisiae*, some pronounced structural features of such landscapes correspond to the mtDNA genome’s origins of replication (*ori*’s). We refer to Zubaer et al. (2019) for illustrations of the rich secondary structures that can be expected for some introns of *nad5*, to Bernardi (2005) for a review of secondary structures in yeasts and other fungi and some of the functional correlates that have been observed for them, and to Misas et al. (2016) for a comparative analysis of oligonucleotide repeat distributions in 11 fungal species with sequenced mitochondrial genomes and a discussion of the phenomenon of heteroplasmy in mitochondrial genomes of fungi. In this last study, *Paracoccidioides* (Pb18, already in the reduced genome of Cardoso et al., 2007) stood out as being one of the most highly repetitive species, yet, among those species, it was characterized by an almost complete preponderance of repeated oligonucleotides (17-mers) that contain no C’s or G’s. This observation suggests not only that the complete assembly of the mtDNA genomes of *Paracoccidioides* spp. is likely to be particularly difficult (as it indeed appears to have been in the past) but also, by the same token, that this genus may be more prone to exhibit structural variants as a result of likely ectopic recombination between very similar, recurring segments and subsequent deletions or excisions of circularizable subsequences.

It might seem unwise to speculate further, in imagining what a possible advantage could be of retaining (at least in one structural variant) the unusually long intron or spacer of *nad5*. This long intron appears to have been present in the mtDNA of both isolates, Pb18 and Pb03, that we sequenced. There could obviously be a disadvantage (as mentioned in a different context by Stearns (1992, p. 201), although possibly with a low energetic expense) in having to replicate, maintain, and/or transcribe long regions of mtDNA (in this case, about 50 kb) that are of no use for the organism. The parallels in *S. cerevisiae*, however, suggest that there might be a benefit in ensuring efficient replication and that, if this is facilitated by structures that the long *nad5* intron can form and that could differ in stability between different temperatures, it could possibly offset maintenance/replication costs. Such a hypothesis could, finally, be considered in the context of the thermal dimorphism of *Paracoccidioides* and other dimorphic Onygenales genera: the organism’s need to function efficiently at two quite different temperature ranges (around 25°C as a saprophyte and around 37°C as a dedicated parasitic yeast), but with only one mtDNA genome sequence at its disposal, would suggest that there might be an advantage in endowing the mtDNA genome with an option to switch between at least two structural variants. However, in the samples we sequenced, none of our experimental observations and *in silico* evidence supported any particular, precise model (DNA sequence) for a likely, more compact variant of the mtDNA genome that does not possess a large *nad5* intron.

From a more general perspective, excessive repetition within a mtDNA genome sequence can result in unfavorable DNA deletion, atypical origins of replication, and chromosomal instability, among other hazards. The permanence of repeats in a mitochondrial genome despite such risks may suggest that some of them could have been favored by selection and may have a structural or functional significance that is still unknown (Misas et al., 2016).

### Mitochondrial Genome Sequences of Onygenales Fungi

In addition to the assemblies of mitochondrial genomes of *Paracoccidioides* spp., there are also available draft assemblies of mitochondrial genomes of other thermally dimorphic fungi within the Onygenales order, such as *Histoplasma capsulatum* (e.g., NCBI accession no. ABBS02000356) and *Blastomyces dermatitidis* (e.g., NCBI accession no. ACBT01000592).

Despite the available information, no comparative studies of the mitochondrial genomes of dimorphic fungi have been conducted. In Onygenales fungi, studies of the comparative genomics at the nuclear level have contributed to an understanding of the different mechanisms of host–pathogen interaction and have facilitated the identification of possible candidate genes for virulence factors (Neafsey et al., 2010; Desjardins et al., 2011; Sharpton, 2014). It is known that in *Paracoccidioides* and other dimorphic fungi, mitochondrial functions play a very important role in the transition from the mycelial to the yeast phase, which is an indispensable step for colonization of the host (Maresca et al., 1981; Medoff et al., 1987; Martins et al., 2008). Studies of mitochondrial genomes can lead to a better understanding of biodiversity and pathophysiology, and of how mitochondrial functions can affect the pathogenicity of this group of dimorphic fungi.

For comparative analyses, the assemblies or annotations of the previously available mitochondrial genomes have unfortunately often been of insufficient quality or have represented only sparsely sampled fungal species. Future analyses of dimorphic fungi should benefit from identification and correction of existing sequence errors or gaps in the available mitochondrial genome assemblies, and from the sequencing and assembly of additional species or strains. In addition to their utility for comparative genomics studies, the new assemblies and annotations of *Paracoccidioides* reported here should be useful as a reliable source for experimental studies, e.g., for investigating how mitochondrial function affects the virulence of *Paracoccidioides* spp.

For such reasons, the availability of optimized mitochondrial sequences such as those reported here responds to a real need of the scientific community. Our updating of the mitochondrial genome assemblies and annotations of the reference strains *P. brasiliensis* Pb18 and *P. americana* Pb03 complements the previously published updating of the corresponding nuclear assemblies for the same strains (Muñoz et al., 2014). The corrected sequences of *Paracoccidioides* spp. are the first genomes of the family Ajellomycetaceae to be revised and optimized in this way, with the aim of providing improved reference sequences for dimorphic fungi.

### Implications for Phylogenetic Analyses

The phylogenetic analysis we have presented was based on the variable positions that were identified in the sequences of the mitochondrial genomes for the 32 isolates of *Paracoccidioides* spp. Although the sizes of the mitochondrial genomes are very much smaller than those of the corresponding nuclear genomes, and thus also have a smaller number of variable sites, those sites have a high confidence because particularly high coverage values were obtained as a result of the depth of sequencing achieved with Illumina. The high coverage is probably largely attributable to the presence of multiple mitochondria per cell, which increases the representation of the mitochondrial genome in the sequencing results.

The genus *Paracoccidioides* includes the species *P. lutzii* and the *brasiliensis* species complex. This main division of the genus *Paracoccidioides* is supported by both nuclear and mitochondrial markers (Matute et al., 2006; Salgado-Salazar et al., 2010; Turissini et al., 2017). However, some lack of convergence has been observed for the grouping of the four species belonging to the *brasiliensis* complex, more specifically between phylogenies recovered from nuclear markers and previous phylogenies based on mitochondrial markers.

Using nuclear sequences, Matute et al. (2006) initially identified three phylogenetic species or lineages within the complex, S1, PS2, and PS3, and the increase of available sequences of *P. brasiliensis* allowed the identification of another group within this species as PS4 (Theodoro et al., 2008; Teixeira et al., 2014), which corresponds to clinical isolates from Venezuela. By contrast, using mitochondrial DNA sequences from five genes, Salgado-Salazar et al. (2010) reported three monophyletic groups, PS2, PS3, and CS1, which corresponds to PS4, but the group S1 (Matute et al., 2006; Turissini et al., 2017) was not recovered and the isolates corresponding to this group were broadly distributed across the tree.

More recently, Turissini et al. (2017) analyzed 12 previously reported loci (five mitochondrial and seven nuclear protein coding genes), together with 10 nuclear non-coding loci and 10 microsatellite loci they sequenced, and proposed that the mito-nuclear incongruence in the *brasiliensis* species complex is the result of interspecific hybridization and mitochondrial introgression. In that report, it was proposed, based on the data then available, that there have been at least three independent mitochondrial gene exchange events between *Paracoccidioides* species (PS3 and S1, PS2 and PS4, and possibly S1 and PS2/PS4). Indeed, the authors found that mitochondrial genes have a less than expected genetic divergence and reported that mitochondrial variation was also more similar between samples from the same geographical region than between samples of the same species, but different regions.

In our phylogenetic reconstruction based on whole mitochondrial SNP analysis of the 32 isolates of the genus *Paracoccidioides* spp., the two isolates of the species *P. lutzii* separate from the others as expected.

Our findings from whole-genome mitochondrial data of the isolates used in Muñoz et al. (2016) confirm an apparent splitting or dispersal of the nuclear genome-based S1 clade of Matute et al. (2006) that was reported when sequences from five loci in the same isolates’ mitochondrial genomes were used instead of nuclear sequences (Salgado-Salazar et al., 2010; Turissini et al., 2017). Although a recent population genomic analysis has characterized a deep subdivision of the *P. brasiliensis* species S1 into two lineages (S1a and S1b) that are distinctly prevalent along eastern Brazil and southern South America, for the isolates we analyzed here we observe three main groups: the S1a group observed by Muñoz et al. (2016) is, in our data, split further into two different clusters (Figure 4). Another finding is that isolates of *P. brasiliensis* S1b, which were denominated as mitochondrial type B21 of S1 in the study by Turissini et al. (2017), appear as the ancestral clade of the *brasiliensis* species complex. Future analyses using nuclear genomes, for example, pooling the isolates we analyzed here with those used in Turissini et al. (2017) and/or other isolates, should help resolve the question of monophyly or paraphyly of S1.

Another feature that has characterized phylogenies constructed from the previously obtained mitochondrial sequences of five loci (Salgado-Salazar et al., 2010; Turissini et al., 2017) was a sister group relationship between PS2 and PS4, which was inconsistent with the isolates’ nuclear phylogenies. In the whole-genome mitochondrial groupings that we find for our set of isolates, PS2 and PS4 do not appear as sister groups (Figure 4). In our reconstruction of relationships in the *brasiliensis* species complex, the taxon *P. venezuelensis* (PS4) tends to group with *P. restrepiensis* (PS3), which would be compatible with both of the nuclear arrangements (Muñoz et al., 2016; Turissini et al., 2017), and confirmation of this finding could reduce the observed nuclear-mitochondrial discrepancies.

## Conclusion

This work presents the update of assemblies and annotations of the mitochondrial genomes of the reference strains *P. brasiliensis* Pb18 and *P. americana* Pb03, complementing the update of the nuclear genomes reported by Muñoz et al. (2014). They are the first mitochondrial sequences of a genus in the Ajellomycetaceae family to be carefully checked and annotated and could therefore be useful as a reference. The phylogenetic reconstruction using the SNPs of the whole mitochondrial genome supports the species differentiation previously described.

## Data Availability Statement

The datasets generated for this study can be found in the NCBI under accession numbers PRJNA322632, SAMN05171520, SRX8563352/SRR12032017, SRX8563354/SRR12032015, SAMN05171542, SRX8563353/SRR12032016, and SRX8563355/SRR12032014.

## Author Contributions

EM, JGM, and OC conceptualized and designed the study. EM and OG assembled, annotated, and analyzed the data of *Paracoccidioides* mitochondrial genomes. EM, OC, OG, VB, MT, JFM, and JGM wrote the manuscript. EM and VB designed and performed the experiments. All authors contributed to the article and approved the submitted version.

## Conflict of Interest

The authors declare that the research was conducted in the absence of any commercial or financial relationships that could be construed as a potential conflict of interest.

## Footnotes

1 https://support.illumina.com/sequencing/sequencing_kits/genomic_dna_sample _prep_kit.html

2 http://lowelab.ucsc.edu/tRNAscan-SE/

3 https://megasun.bch.umontreal.ca/RNAweasel/

## Supplementary Material

FIGURE S1 Mitochondrial genome assembly showing in yellow the insert, i.e., the region absent in the mitochondrial genome Pb18 assembled by Cardoso et al. (2007), in dark green the forward primers and in light green the reverse primer for the two amplicons in flanking regions.

FIGURE S2 PCR amplicons on a 1.2% agarose gel electrophoresis A. for Mito1 primers and B. for Mito2 primers.

TABLE S1 Primer sequences used to determine presence/absence of the insert.

TABLE S2 Summary characteristics of the de novo assemblies obtained using Canu v-1.5 and SPAdes v-3.10.
