# Supplementary material for: Updates and Comparative Analysis of the Mitochondrial Genomes of Paracoccidioides spp. Using Oxford Nanopore MinION Sequencing
Source: Front Microbiol. 2020 Aug 4;11:1751. doi: 10.3389/fmicb.2020.01751 (PMC7417371; doi:10.3389/fmicb.2020.01751)
Supplement: FIGURE S1 — Mitochondrial genome assembly showing in yellow the insert, i.e., the region absent in the mitochondrial genome Pb18 assembled by Cardoso et al. (2007), in dark green the forward primers and in light green the reverse primer for the two amplicons in flanking regions. [file Table_3.docx]

**Supplementary Material:**

***In vitro* analysis to confirm the insert region in the mitochondrial genome of *Paracoccidioides* spp.**

In the mitochondrial genome assemblies obtained using Oxford Nanopore reads, a region or “insert” was identified that is absent in the mitochondrial genome sequences previously reported. This region has a length of 47 kb in the Pb18 assembly and 38 kb in the Pb03 assembly.

To confirm the presence of the insert region on the mitochondrial genome assembly of Pb18, we designed two pairs of primers (Table S3) in the flanking region of the “insert” of the mitochondrial genome assembly of Pb18 (Figure S1.1).

In addition to the reference isolate *P. brasiliensis* (Pb18), we analyzed mitochondrial DNA of the isolates *P. americana* (Pb03), *P. restrepiensis* (Pb60855), *P. venezuelensis* (Pb300) and *P. lutzzi* (Pb01), in order to investigate presence/absence of the “insert” in the different species of the *Paracoccioides* genus. Each isolate was evaluated in duplicate.

The PCR was performed in a 20 μl reaction mixture containing 1 µL of genomic DNA, 0.4 µL dNTPs, 0.4 µL primers (forward and reverse), 2 µL Taq buffer with KCl and (NH4)2SO4, 0.3 µL Taq DNA Polymerase (Thermo scientific), and water free of nucleases. The concentration of MgCl_2_ was variable, due to the fact that a magnesium gradient between 2.0M- 3.5M was used to identify the optimal concentration for the amplification.

The amplification conditions were as follows: one cycle at 94°C for 45 min; 35 cycles at 94°C for 40 s, 52°C to 58°C (temperature gradient) for 30 s, 72°C for 45 s; and a final extension cycle at 72°C for 5 min. In all of the PCR assays, 1 μl of water free of nucleases was processed as the negative control and 1 μl of TUB1 gene was processed as the positive control.

The selected conditions were different for each of the two pairs of primers. The melting temperature and magnesium concentration were standardized. The best amplification conditions for MITO1 were 57.5°C and 2.0 mM MgCl_2_ ; MITO2 58.0°C and 2.0 mM MgCl_2_ The amplified products were resolved by electrophoresis in 1.2% agarose gels in 0.5X Tris-borate-EDTA buffer at 80V (Figure S1.2).

Using MITO1 primers, PCR products were amplified in all the isolates (Figure S1.2A) and using MITO2 primers, PCR products were amplified from *P. brasiliensis* (Pb18), *P. restrepiensis* (Pb60855) and *P. venezuelensis* (Pb300; Figure S1.2B). The absence of amplicons for the isolates *P. lutzi* (Pb01) and *P. americana* (Pb03) might be due to changes in the nucleotide sequence of the left flanking region of the “insert” of the hese isolates, as the primers were designed based on Pb18 mitochondrial genome assembly.

According to the PCR results we can conclude that the insert region is present in the mitochondrial genome of the three species of *Paracoccidioides* spp.; *P. brasiliensis* (Pb18), *P. restrepiensis* (Pb60855) and and *P. venezuelensis* (Pb300. For *P. americana* we found in *in silico* evidence that support the presence of the “insert”, but this could not be fully confirmed in the in vitro analyzes.

*
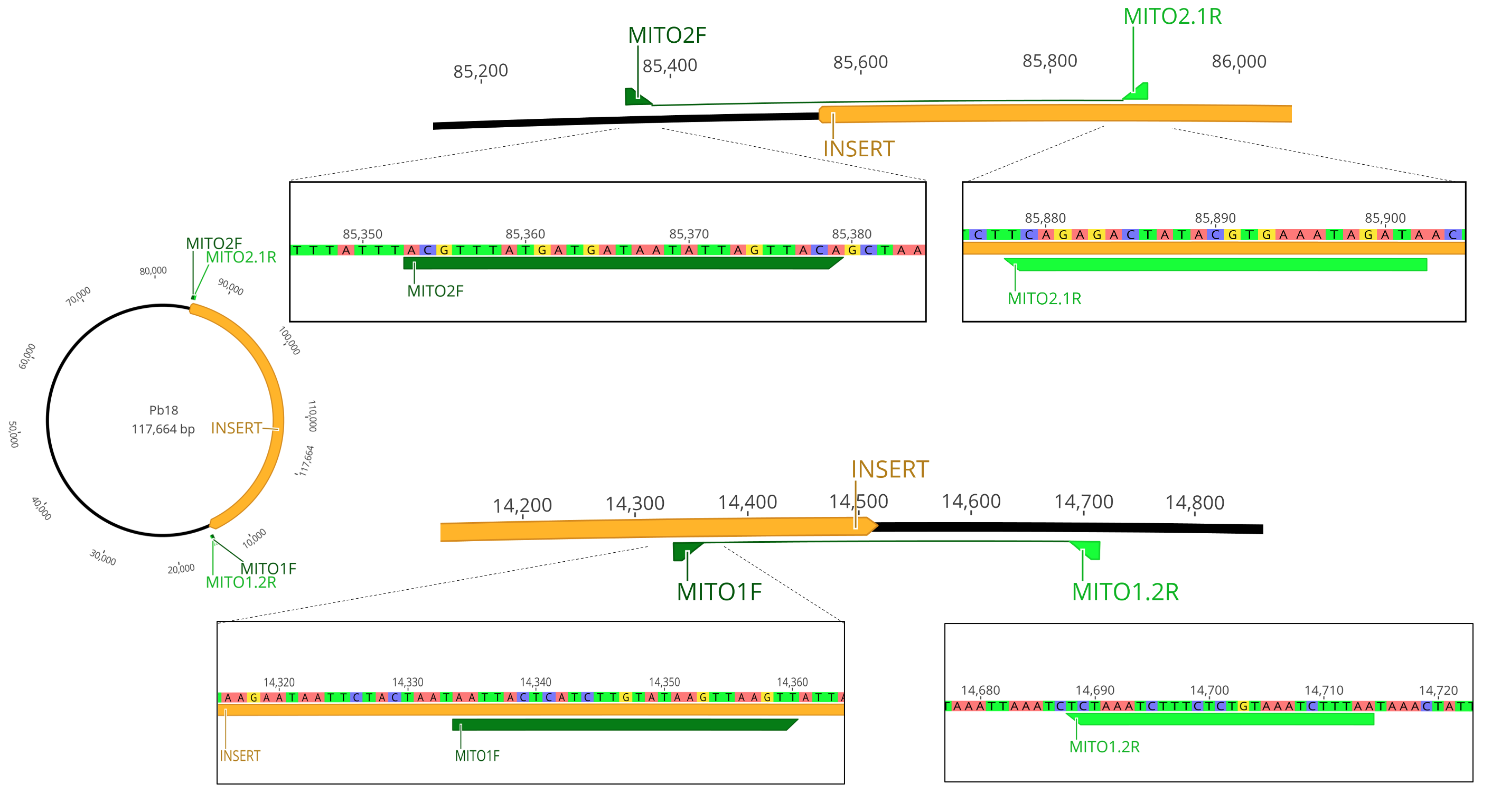
*

**Figure S1.1:** Mitochondrial genome assembly showing in yellow the insert, the region absent in the mitochondrial genome Pb18 assembly by Cardoso et al (2007), in dark green the forward primers and in light green the reverse primer for the two amplicons in flanking regions.

*
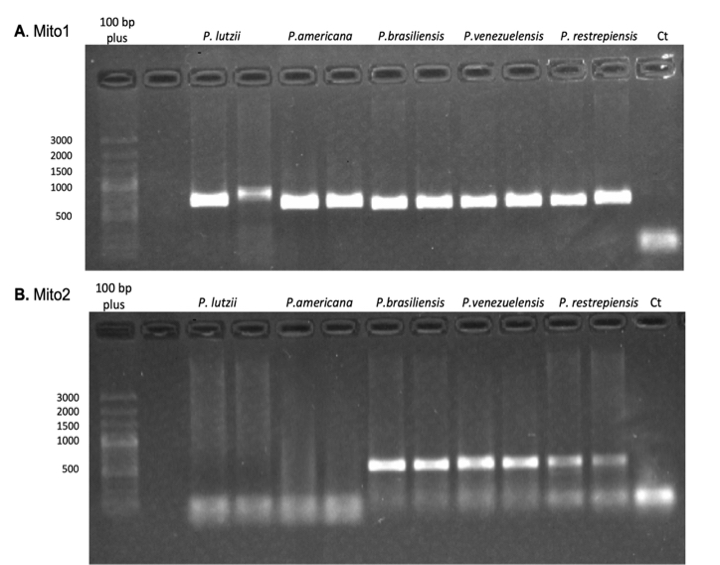
*

**Figure S1.2:** PCR amplicons on a 1.2% agarose gel electrophoresis (**A**) for Mito1 primers and (**B**) for Mito2 primers.

**Supplementary Table S3:** Primer sequences used to determine presence/absence of the insert.

| **Primer name** | **Direction** | **Sequence** |
| --- | --- | --- |
| MITO2F | Forward | ACGTTTATGATGATAATATTAGTTACA |
| MITO2.2R | Reverse | ATCTATTTCACGTATAGTCTCTGAAGA |
| MITO2.1R | Reverse | TATCTATTTCACGTATAGTCTCTGA |
| MITO1R | Reverse | AGTTTATTAAAGATTTACAGAGAAAGA |
| MITO1F | Forward | AATTACTCATCTTGTATAAGTTAAGTT |
| MITO1.2R | Reverse | TTAAAGATTTACAGAGAAAGATTTAGA |
