# Supplementary material for: Updates and Comparative Analysis of the Mitochondrial Genomes of Paracoccidioides spp. Using Oxford Nanopore MinION Sequencing
Source: Front Microbiol. 2020 Aug 4;11:1751. doi: 10.3389/fmicb.2020.01751 (PMC7417371; doi:10.3389/fmicb.2020.01751)
Supplement: TABLE S2 — Summary characteristics of the de novo assemblies obtained using Canu v-1.5 and SPAdes v-3.10. [file Table_2.docx]

**Supplementary Table S2:** Summary characteristics of the *de novo* assemblies obtained using Canu v-1.5 and SPAdes v-3.10
